# Association of Diabetes Mellitus With Severity Complications and Mortality in Acute Pancreatitis Patients

**DOI:** 10.7759/cureus.110643

**Published:** 2026-06-11

**Authors:** Adnan Ur Rehman, Sahrish Naz, Muhammad Kashif Rafiq, Kamal Uddin Azam, Nusratullah Khan, Abdul Wasay Javed

**Affiliations:** 1 Gastroenterology and Hepatology, Hayatabad Medical Complex Peshawar, Peshawar, PAK; 2 Medicine, i-Care Medical Center, Imran Idrees Teaching Hospital, Sialkot, PAK; 3 General Surgery, Ayub Teaching Hospital Abbottabad, Abbottabad , PAK; 4 Surgery, University Hospital of North Durham, Durham, GBR; 5 Biochemistry, Mekran Medical College Turbat, Turbat, PAK; 6 Physiology, M. Islam Medical and Dental College, Gujranwala, PAK

**Keywords:** acute pancreatitis, diabetes mellitus, mortality, organ failure, pancreatic necrosis

## Abstract

Background: Acute pancreatitis is a common inflammatory disease with variable clinical outcomes. Diabetes mellitus may worsen the severity and increase complications in these patients. This study was conducted to evaluate the association of diabetes mellitus with severity, complications, and mortality in acute pancreatitis patients.

Methods: This prospective observational study was conducted at Ayub Medical College, Abbottabad from April 2025 to September 2025. A total of 160 patients with confirmed acute pancreatitis were included through consecutive sampling. Patients were divided into diabetic and non-diabetic groups. Severity was assessed by the Revised Atlanta Classification. Demographic profile, laboratory findings, complications, ICU admission, hospital stay, and mortality were recorded. Data were analyzed using IBM SPSS Statistics for Windows, Version 26 (Released 2018; IBM Corp., Armonk, New York, United States). The chi-square test, independent sample t-test, and multivariable logistic regression analysis were applied. A p-value less than 0.05 was considered significant.

Results: Out of 160 patients, 68 (42.5%) were diabetic, and 92 (57.5%) were non-diabetic. Severe acute pancreatitis was significantly more common in diabetic patients (30.9% vs 15.2%, p=0.017). Persistent organ failure (27.9% vs 13.0%, p=0.019), pancreatic necrosis (25.0% vs 12.0%, p=0.031), ICU admission (29.4% vs 14.1%, p=0.018), and mortality (16.2% vs 8.7%, p=0.041) were also higher among diabetic patients. Mean hospital stay was significantly prolonged in diabetics (8.7±3.9 vs 5.9±2.8 days, p<0.001). Multivariable analysis showed diabetes mellitus as an independent predictor of severe acute pancreatitis and mortality.

Conclusion: Diabetes mellitus was significantly associated with increased severity, complications, prolonged hospital stay, and mortality in acute pancreatitis. Early identification of diabetic patients may help improve clinical outcomes.

## Introduction

Acute pancreatitis is an inflammatory condition of the pancreas that can range from mild self-limiting disease to severe life-threatening illness [1]. The incidence of acute pancreatitis is increasing worldwide due to rising obesity, gallstones, alcohol use, and metabolic disorders [2]. Most patients recover with supportive treatment, but some develop organ failure, pancreatic necrosis, sepsis, and death [3].

Diabetes mellitus is one of the most common chronic diseases globally [4]. It affects multiple organs through chronic inflammation, endothelial dysfunction, impaired immunity, and metabolic disturbance [5,6]. These changes may worsen the course of acute inflammatory diseases including acute pancreatitis. Several studies have shown that diabetic patients may experience more severe pancreatitis, prolonged hospital stay, and increased mortality compared to non-diabetic patients [6,7].

The exact relationship between diabetes mellitus and acute pancreatitis is still not fully understood. Chronic hyperglycemia can impair pancreatic microcirculation and increase inflammatory cytokine release [8]. This may increase the risk of pancreatic necrosis and persistent organ failure [9]. Diabetes is also commonly associated with obesity, hypertension, and hypertriglyceridemia, which themselves are known risk factors for severe pancreatitis [10].

Previous international studies reported that diabetic patients had higher rates of ICU admission, local complications, and death during acute pancreatitis [11]. A population-based study found that diabetes increased the risk of severe acute pancreatitis and hospital mortality [12]. Another recent study also identified diabetes mellitus as an independent predictor of severe pancreatitis and organ failure [11].

Despite increasing evidence, local Pakistani data on this topic remain limited. Most available studies were conducted in Western populations with different demographic and metabolic profiles. Due to the increasing burden of diabetes in Pakistan, understanding its effect on acute pancreatitis is important for early risk stratification and management.

Therefore, the present study was conducted to determine the association of diabetes mellitus with severity, complications, and mortality in patients with acute pancreatitis admitted to a tertiary care hospital in Kohat, Pakistan.

## Materials and methods

This hospital-based prospective observational study was conducted at Ayub Medical College, Abbottabad, Pakistan over a period of six months from April 2025 to September 2025. The study was designed and reported according to the STROBE guidelines for observational studies [13]. Ethical approval was obtained from the Institutional Research and Ethics Committee of the Ayub Medical College, Abbottabad before data collection (Reference No. 2109/25; dated April 19, 2025). Written informed consent was obtained from all patients or their attendants.

A total sample size of 160 patients was calculated using OpenEpi software by taking 95% confidence level, 80% study power, expected frequency of diabetes mellitus among patients with acute pancreatitis of 21.3%, as reported in a previous study [11], and a margin of error of 5%. A total of 166 eligible patients were screened during the study period. Six patients were excluded because of incomplete clinical or laboratory records. Therefore, 160 patients were included in the final analysis. Patients were selected through non-probability consecutive sampling. Adult patients aged 18 years or above with a confirmed diagnosis of acute pancreatitis were included. Diagnosis was made on the basis of at least two of the following criteria: characteristic abdominal pain, serum amylase or lipase level more than three times the upper normal limit, and radiological findings suggestive of acute pancreatitis on ultrasonography or CT scan. The detailed inclusion and exclusion criteria are presented in Table 1.

**Table 1:** Inclusion and exclusion criteria of the study participants

Inclusion Criteria	Exclusion Criteria
Patients aged 18 years or above	Patients with chronic pancreatitis
Patients with a confirmed diagnosis of acute pancreatitis based on revised Atlanta criteria	Patients with recurrent acute pancreatitis, active malignancy, current immunosuppressive therapy, and pancreatogenic (type 3C) diabetes
Patients admitted during the study period	Pregnant women
Patients willing to participate in the study	Trauma-related pancreatitis
Patients with complete clinical and laboratory records	Patients with chronic liver disease
	Patients with chronic kidney disease
	Patients with autoimmune disorders
	Patients with incomplete medical records

Patients were divided into diabetic and non-diabetic groups according to the American Diabetes Association (ADA) criteria [14]. Patients were classified as diabetic if they had a previous physician diagnosis of diabetes mellitus, were receiving anti-diabetic treatment, had a fasting plasma glucose level ≥126 mg/dL (7.0 mmol/L), or an HbA1c level ≥6.5%. Demographic data, body mass index, smoking history, etiology of pancreatitis, laboratory findings, hospital stay, local complications, organ failure, intensive care unit admission, and in-hospital mortality were recorded on a structured proforma. Severity of acute pancreatitis was assessed prospectively throughout hospitalization using the Revised Atlanta Classification. The final severity category was assigned after evaluation of the complete clinical course, including the presence and duration of organ failure and local complications. Persistent organ failure lasting more than 48 hours was considered severe disease.

Possible confounders including age, gender, obesity, smoking, gallstones, alcohol use, hypertriglyceridemia, and hypertension were recorded and adjusted during statistical analysis. Alcohol-related pancreatitis was recorded during data collection; however, due to its very low frequency in the study population, it was not retained in the final multivariable regression model. A small number of patients were excluded because of missing clinical or laboratory data, which may have introduced a degree of selection bias. However, the number of excluded cases was low and is unlikely to have materially affected the study findings.

Data were analyzed using IBM SPSS Statistics for Windows, Version 26 (Released 2018; IBM Corp., Armonk, New York, United States). Quantitative variables were presented as mean and standard deviation, while qualitative variables were presented as frequency and percentage. The chi-square test and independent sample t-test were applied where appropriate. Multivariable logistic regression analysis was performed to determine the independent association of diabetes mellitus with severity, complications, and mortality in acute pancreatitis after adjustment of confounding variables. A p-value of less than 0.05 was considered statistically significant.

## Results

A total of 160 patients with acute pancreatitis were included in the final analysis. Among them, 68 (42.5%) patients had diabetes mellitus, while 92 (57.5%) patients were non-diabetic. The mean age of the study population was 49.8±13.7 years. Diabetic patients were relatively older and had higher body mass index compared to non-diabetic patients. Gallstones were the most common cause of acute pancreatitis in both groups. Baseline demographic and clinical characteristics are shown in Table 2. 

**Table 2:** Baseline demographic and clinical characteristics of study participants An independent sample t-test was used for quantitative variables, and the chi-square (χ²) test was used for qualitative variables. SD: Standard deviation; BMI: body mass index; U/L: units per liter.

Variables	Diabetic (n=68)	Non-diabetic (n=92)	Test statistic	p-value
Age (years), Mean ± SD	54.6 ± 11.8	46.2 ± 14.1	t(158) = 4.08	0.001
Male gender, n (%)	40 (58.8%)	49 (53.3%)	χ²(1) = 0.48	0.489
BMI (kg/m²), Mean ± SD	29.1 ± 4.2	25.8 ± 3.7	t(158) = 5.23	<0.001
Smokers, n (%)	24 (35.3%)	27 (29.3%)	χ²(1) = 0.65	0.421
Hypertension, n (%)	39 (57.4%)	18 (19.6%)	χ²(1) = 24.71	<0.001
Gallstone pancreatitis, n (%)	36 (52.9%)	46 (50.0%)	χ²(1) = 0.13	0.716
Hypertriglyceridemia, n (%)	18 (26.5%)	11 (12.0%)	χ²(1) = 5.61	0.018
Serum amylase (U/L), Mean ± SD	742 ± 291	701 ± 266	t(158) = 0.92	0.356
Serum lipase (U/L), Mean ± SD	911 ± 348	864 ± 331	t(158) = 0.84	0.401

Severity assessment showed that severe acute pancreatitis was more common in diabetic patients. Persistent organ failure, pancreatic necrosis, and ICU admission were also significantly higher among diabetic patients as shown in Table 3.

**Table 3:** Severity and complications of acute pancreatitis in both groups The chi-square (χ²) test was used for categorical variables, and the independent sample t-test was used for hospital stay duration. ICU: Intensive care unit; SD: standard deviation

Variables	Diabetic (n=68)	Non-diabetic (n=92)	Test statistic	p-value
Mild acute pancreatitis, n (%)	21 (30.9%)	49 (53.3%)	χ²(1) = 7.74	0.005
Moderately severe pancreatitis, n (%)	26 (38.2%)	29 (31.5%)	χ²(1) = 0.78	0.376
Severe acute pancreatitis, n (%)	21 (30.9%)	14 (15.2%)	χ²(1) = 5.67	0.017
Persistent organ failure, n (%)	19 (27.9%)	12 (13.0%)	χ²(1) = 5.49	0.019
Pancreatic necrosis, n (%)	17 (25.0%)	11 (12.0%)	χ²(1) = 4.66	0.031
Acute respiratory distress syndrome, n (%)	11 (16.2%)	7 (7.6%)	χ²(1) = 2.87	0.091
Acute kidney injury, n (%)	14 (20.6%)	8 (8.7%)	χ²(1) = 4.70	0.030
ICU admission, n (%)	20 (29.4%)	13 (14.1%)	χ²(1) = 5.57	0.018
Mean hospital stay (days)	8.7 ± 3.9	5.9 ± 2.8	t(158) = 5.20	<0.001

Mortality was significantly higher in diabetic patients compared to non-diabetic patients. Overall, in-hospital mortality was 19 (11.9%; Table 4).

**Table 4:** Clinical outcomes and mortality among study participants The chi-square (χ²) test was used for all categorical outcome variables. Mortality was recorded during hospital admission only.

Variables	Diabetic (n=68)	Non-diabetic (n=92)	Test statistic	p-value
Recovered and discharged, n (%)	57 (83.8%)	84 (91.3%)	χ²(1) = 2.09	0.148
In-hospital mortality, n (%)	11 (16.2%)	8 (8.7%)	χ²(1) = 4.17	0.041
Septic complications, n (%)	15 (22.1%)	10 (10.9%)	χ²(1) = 3.86	0.049
Need for ventilatory support, n (%)	13 (19.1%)	7 (7.6%)	χ²(1) = 4.84	0.028
Shock requiring vasopressors, n (%)	12 (17.6%)	6 (6.5%)	χ²(1) = 4.97	0.026

Potential confounders including age, gender, smoking status, obesity, hypertension, gallstone etiology, and alcohol use were initially evaluated. Variables showing limited variability or no meaningful association with study outcomes were excluded from the final regression model. The final adjusted model included age, gender, smoking status, obesity, hypertension, and hypertriglyceridemia. Diabetes mellitus remained independently associated with severe acute pancreatitis, pancreatic necrosis, and mortality (Table 5).

**Table 5:** Multivariable logistic regression analysis for predictors of severe outcomes Multivariable logistic regression analysis was performed after adjustment for age, gender, smoking, obesity, hypertension, and hypertriglyceridemia. AOR: Adjusted odds ratio; ICU: intensive care unit; CI: confidence interval

Variables	Adjusted Odds Ratio (AOR)	95% Confidence Interval	Wald statistic	p-value
Diabetes mellitus and severe acute pancreatitis	2.64	1.28 – 5.41	6.98	0.008
Diabetes mellitus and pancreatic necrosis	2.31	1.04 – 5.12	4.27	0.039
Diabetes mellitus and ICU admission	2.18	1.05 – 4.52	4.45	0.035
Diabetes mellitus and mortality	2.72	1.01 – 7.29	3.93	0.047
Obesity and severe pancreatitis	1.89	1.02 – 3.51	4.12	0.042
Hypertriglyceridemia and pancreatic necrosis	2.11	1.01 – 4.43	3.98	0.046

## Discussion

Acute pancreatitis is a common emergency disease, but its outcome becomes more serious when the patient already has diabetes mellitus [15]. In our study, diabetic patients had more severe disease, more complications, longer hospital stay, more ICU admissions, and higher mortality. This finding is clinically important because diabetes is common in Pakistan, and early risk assessment can improve patient care.

In the present study, diabetic patients were older and had higher BMI and hypertension. These factors can increase the stress on the body during acute pancreatitis. Similar findings were reported in previous studies where diabetes, obesity, and other comorbid diseases were linked with poor outcomes and ICU need in acute pancreatitis [16]. A large study also reported that diabetic patients with acute pancreatitis had a higher risk of ICU admission and local complications [17].

Severe acute pancreatitis was more frequent in diabetic patients in our study. This may be due to chronic hyperglycemia, weak immune response, endothelial dysfunction, and increased inflammatory activity in diabetes [18]. These changes can worsen pancreatic injury and delay recovery. A recent study also showed that diabetes increased the risk of moderate to severe pancreatitis and mortality [13].

Our diabetic patients also showed more pancreatic necrosis, persistent organ failure, and acute kidney injury. This is understandable because diabetes affects small blood vessels and reduces tissue healing. Poor microcirculation may increase pancreatic ischemia and necrosis [19]. Organ failure is a major sign of severe pancreatitis according to the Revised Atlanta Classification [20]. Persistent organ failure is strongly linked with worse outcomes and death [21].

ICU admission and ventilatory support were also higher in diabetic patients. This supports the idea that diabetic patients develop more systemic complications. A nationwide inpatient study found diabetes to be an independent risk factor for ICU admission in acute pancreatitis [22].

Mortality was higher in diabetic patients in our results. This agrees with published evidence showing that type 2 diabetes is associated with more severe acute pancreatitis and increased mortality. One study reported about 58% higher ICU admission risk and 30% higher local complication risk among diabetic patients [23].

Clinically, these findings suggest that diabetic patients with acute pancreatitis should be monitored more carefully from admission. Early fluid resuscitation, glucose control, renal monitoring, infection control, and timely ICU referral may reduce complications [24].

The main limitations of this study were a single-center design, a limited sample size, and short follow-up. Long-term outcomes after discharge were not assessed. Alcohol use was recorded during data collection; however, alcohol-related pancreatitis was uncommon in our study population. Therefore, its effect on disease severity and outcomes could not be evaluated separately. Future multicenter studies involving more diverse populations may better assess the influence of alcohol-related pancreatitis on clinical outcomes.

## Conclusions

Diabetes mellitus was significantly associated with severe acute pancreatitis, pancreatic necrosis, organ failure, ICU admission, prolonged hospital stay and mortality. Diabetic patients should be considered a high-risk group in acute pancreatitis.
